# Electronic health records research: easy evidence, harder oversight

**DOI:** 10.1093/ehjdh/ztag105

**Published:** 2026-07-02

**Authors:** Abdalhakim Shubietah, Kerollos Abdelsayed, Mohammed Ruzieh

**Affiliations:** Department of Medicine, Advocate Illinois Masonic Medical Center, Chicago, IL, USA; Heart Rhythm Science Center, Minneapolis Heart Institute Foundation, Minneapolis, MN, USA; Bluhm Cardiovascular Institute, Feinberg School of Medicine, Northwestern University, 675 N St Clair, Chicago, IL 60611, USA

**Keywords:** Electronic health records, Real-world evidence, Aggregate data, Research networks, Reproducibility

## Abstract

Electronic health record (EHR) research networks have accelerated clinical research by making it possible to study large and diverse patient populations over time. However, the quality and resources required for these studies vary widely, depending on whether they rely on simple aggregate outputs or a more rigorous patient-level data, a central but occasionally unstated distinction in EHR-based research. Patient-level data can support a more reproducible cohort construction, time-anchored exposure definitions, and more precise outcome ascertainment, whereas aggregate approaches limit rigour regarding the precise definition of index dates, the completeness of covariate capture, the accurate characterization of medication exposure windows, and the validity of outcome ascertainment. These limitations may amplify well-known threats to causal inference in observational research, including misclassification, incomplete outcome capture across health systems, residual confounding, and immortal time bias, particularly when comparative effectiveness questions are pursued. In this viewpoint, we outline why ‘easy evidence’ can yield fragile conclusions when analytic granularity is limited, and we propose pragmatic expectations for reporting and review: explicit declaration of data level (patient vs. aggregate), clear time-zero definitions for all arms, validated case-definition algorithms when available, sensitivity analyses aligned with plausible misclassification, and transparency sufficient to enable reproducibility. Strengthening methodological norms is essential to preserve the credibility of EHR-based evidence as its influence on clinical discourse continues to grow.

## Graphical Abstract

**Graphical Abstract:**
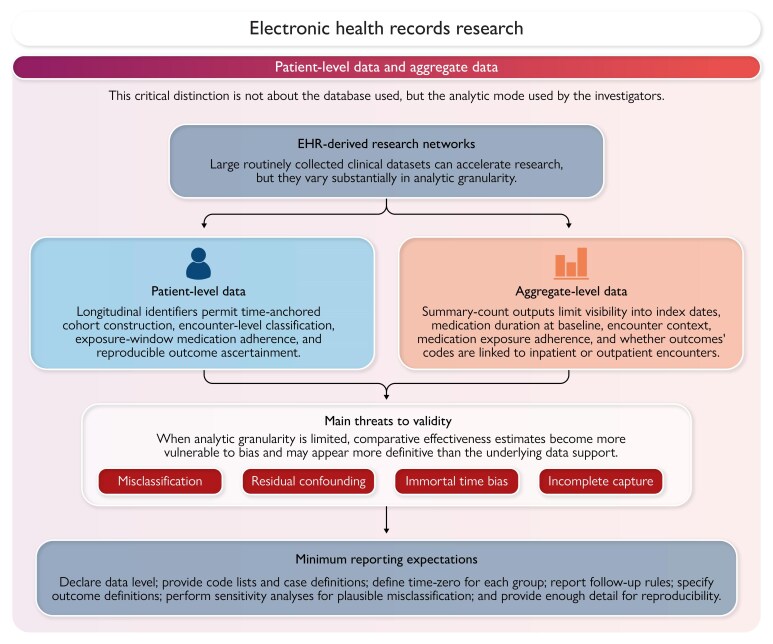

Electronic health record (EHR) derived research networks have transformed clinical research by allowing researchers to easily access pre-existing data that are collected routinely for clinical care rather than research purposes. However, these networks are not all the same. Some provide patient-level data with extensive data harmonization (such as the Electronic Medical Records and Genomics ‘eMERGE’ network and PCORnet), whereas others are commercially available query platforms where investigators can use built-in tools to define cohorts and generate aggregate results without direct access to the underlying patient-level data.

EHR data offer several important advantages for clinical and outcomes research. As these data are collected routinely during real-world clinical care, they reflect everyday practice across diverse patient populations, enhancing generalizability. EHRs capture large sample sizes with longitudinal follow-up, allowing researchers to study rare exposures, uncommon outcomes, and long-term disease trajectories. Furthermore, EHR-based studies are relatively time- and cost-efficient compared with prospective studies, facilitating rapid hypothesis testing, and allowing evaluation of treatment effectiveness, safety, and healthcare utilization in real-world settings.

There are several large-scale databases including Medicare claims data, TriNetX and COSMOS. Publications leveraging TriNetX and COSMOS datasets have increased markedly over the past several years, from a single publication in 2018 to more than 2500 publications to date, with the vast majority from TriNetX, and predominantly using *aggregate-data.*

In contrast to Medicare claims data, which provides only patient-level data, platforms such as TriNetX and Epic COSMOS offer patient-level data but also offer access to aggregate-level analyses. This distinction is critically important for investigators, peer reviewers, and readers, as it has implications for study design, analytic transparency, interpretation of results, and reproducibility. When investigators do not have access to the underlying patient-level longitudinal data, or when the platform does not clearly report how exposures, covariates, index dates, follow-up windows, and outcomes were defined, the resulting estimates may be difficult to interpret. This concern is especially important when the primary outcome is based on administrative codes or platform-defined algorithms that cannot be independently audited. In such cases, aggregate outputs may obscure whether an event represents a clinically meaningful outcome, a historical diagnosis, an incidental code, or a billing artifact.

When using patient-level data, such as Medicare claims data, each individual is assigned a unique identifier that enables longitudinal tracking of medical history using ICD codes and medications, medication use, procedures using CPT codes, and clinical outcomes over time. This structure allows investigators to apply rigorous and reproducible definitions for baseline comorbidities and outcomes. For example, diabetes can be defined using strict criteria such as an inpatient admission with diabetes listed as a primary or secondary diagnosis, two outpatient encounters coded for diabetes, or documented insulin use within the year preceding the index date. Similarly, outcomes such as heart failure can be defined as an inpatient hospitalization in which heart failure or heart failure exacerbation is recorded as the primary diagnosis. This level of granularity enhances the accuracy of cohort construction and outcome ascertainment. In contrast, aggregate data, do not permit the same level of clinical granularity or rigor in exposure and outcome definitions. In aggregate analyses, investigators have access to only summarized counts generated by the software, without visibility into individual-level longitudinal data, and this is a very important distinction. As a result, exposures are typically defined using ICD codes alone, without the ability to determine whether a diagnosis occurred in the inpatient or outpatient setting and whether it represented an active clinical condition or a historical diagnosis. This limitation is particularly consequential given that ICD codes demonstrate less than 60% accuracy in EHR-based data.^1,2^ Without access to patient-level data to reliably track inpatient and outpatient encounters, as well as medication use related to the diagnosis, the validity of the resulting estimates is substantially compromised.

More importantly, outcome ascertainment is substantially limited. Aggregate data do not allow investigators to distinguish between clinically meaningful events and administrative coding artifacts. For instance, heart failure will be counted as an outcome if it appears in any inpatient or outpatient encounter, without the ability to determine whether it reflects an acute decompensation requiring hospitalization, a historical diagnosis carried forward, or a code used incidentally during testing or billing. For example, when studying heart failure exacerbations as an outcome, researchers may rely on proxy codes such as ‘acute systolic heart failure’ or ‘acute diastolic heart failure’; however, these codes lack sensitivity and specificity and do not reliably distinguish true acute decompensations from historical or incidental diagnoses.^3^ Similarly, when the ICD code I63 is used to define ischemic stroke, it does not tell us if that was an acute or historic diagnosis.^4^ Consequently, clinically important outcomes such as heart failure exacerbation cannot be reliably defined, yet are frequently reported as endpoints in aggregate-data–based studies. This limitation significantly compromises the validity of outcome assessment in aggregate datasets.

To illustrate these two points concretely, consider an EHR-based study examining the effect of drug X on the risk of myocardial infarction, comparing patients who received the drug with those who did not, with hypertension and diabetes included as baseline covariates. In a patient-level analysis, hypertension can be defined with precision; for example, by requiring at least two outpatient encounters carrying a hypertension ICD code, or documentation of antihypertensive medication use for a minimum of six months prior to the index date. The same granular definition can be applied to diabetes. This level of specificity matters because a single mention of hypertension in one outpatient encounter may reflect a transient finding, a data-entry error, or a rule-out diagnosis rather than an established condition, and a medication prescribed for one week cannot be treated as equivalent to a medication used continuously for months when adjusting for confounding. In aggregate data, this precision is not currently achievable; the investigator can instruct the software to control for hypertension, diabetes, and medication use at baseline, but the lack of granularity leaves confounders that cannot be fully characterized or mitigated.

The same limitation applies to outcome ascertainment. In patient-level data, myocardial infarction can be defined using ICD codes if an inpatient encounter lists myocardial infarction as the primary diagnosis, or as a secondary diagnosis only when the primary diagnosis represents a complication of myocardial infarction, a definition that excludes type 2 myocardial infarction occurring in the context of a separate critical illness such as severe sepsis. In aggregate data, any encounter carrying a myocardial infarction diagnostic code may be counted as an outcome event regardless of clinical context, whether it was mentioned in an inpatient or outpatient encounter, or whether it represents an old or new diagnosis. While type 2 myocardial infarction ICD codes can be excluded in aggregate approaches, this method is imprecise and introduces its own misclassification uncertainty, which patient-level phenotyping algorithms are specifically designed to avoid.

Another issue with studies using EHR-based data sources, such as TriNetX, compared with insurance claims data such as Medicare, is the potential for incomplete outcome capture, as EHR datasets may miss clinical events or deaths that occur outside the participating health care networks.

Another important concern is immortal time bias, which is already difficult to control in observational studies and becomes even more challenging when using aggregate data since authors only get summary counts. In such datasets, defining an appropriate index date, particularly for the control arm, is often problematic. For example, when comparing outcomes after an intervention vs. no intervention, aggregate outputs may not clearly specify when follow-up begins for the non-intervention group.

Another important limitation of aggregate data relates to the assessment of baseline medication use. The available aggregate data lack sufficient granularity to reliably determine true medication exposure at baseline, making it difficult to distinguish between a single prescription, inpatient administration during hospitalization, or sustained outpatient use over time. As a result, baseline medication status is frequently misclassified, limiting the validity of medication-based adjustments and introducing substantial residual confounding.

Using retrospective EHR data to evaluate trends and health care utilization can be informative; however, conducting comparative effectiveness analyses is substantially more challenging, and while some studies are well conducted and have low risk for bias, the results from many studies do not align with randomized clinical trials. For example, most observational studies assessing beta-blockers in patients with chronic obstructive pulmonary disease showed significant benefit with beta-blockers but when the question was tested in a clinical trial, beta-blockers were associated with harm.^5^ Another example is the effect of hormone replacement therapy on cardiovascular disease in postmenopausal women where observational studies suggested benefit, but trial data showed harm.^6,7^ These studies used patient-level observational data and reported results discordant to those seen in clinical trials. This discrepancy likely reflects residual confounding and immortal time bias, which is difficult to fully address in observational analyses. If patient-level data can fail to produce accurate treatment effect estimates, the potential for bias is even greater when relying on aggregate-level data.

Accordingly, studies using EHR-data should clearly report the level of data used (aggregate vs. patient-level data), the code lists and case definitions used, the time-zero definition for each study group, follow-up, and definition of outcomes.

## Data Availability

No data was generated or analyzed for or in support of this paper.

## References

[1] Zelicha H, Bell DS, Chen Y, Livingston EH. Potential for error when relying on administrative data. Br J Surg 2025;112:Znaf139. 40673449 10.1093/bjs/znaf139 PMC12268322

[2] Voss RW, Schmidt TD, Weiskopf N, Marino M, Dorr DA, Huguet N, et al Comparing ascertainment of chronic condition status with problem lists versus encounter diagnoses from electronic health records. J Am Med Inform Assoc 2022;29:770–778. 35165743 10.1093/jamia/ocac016 PMC9006679

[3] Bates BA, Akhabue E, Nahass MM, Mukherjee A, Hiltner E, Rock J, et al Validity of International Classification of Diseases (ICD)-10 diagnosis codes for identification of acute heart failure hospitalization and heart failure with reduced versus preserved ejection fraction in a national medicare sample. Circ Cardiovasc Qual Outcomes 2023;16:e009078. 36688301 10.1161/CIRCOUTCOMES.122.009078

[4] McCormick N, Bhole V, Lacaille D, Avina-Zubieta JA. Validity of diagnostic codes for acute stroke in administrative databases: a systematic review. PLoS One 2015;10:e0135834. 26292280 10.1371/journal.pone.0135834 PMC4546158

[5] Ruzieh M, Baugh AD, Al Jebbawi L, Edwards ES, Jia KQ, Dransfield MT, et al Beta-blocker use in patients with chronic obstructive pulmonary disease: a systematic review: a systematic review of βB in COPD. Trends Cardiovasc Med 2023;33:53–61. 34856338 10.1016/j.tcm.2021.11.004

[6] Grady D, Rubin SM, Petitti DB, Fox CS, Black D, Ettinger B, et al Hormone therapy to prevent disease and prolong life in postmenopausal women. Ann Intern Med 1992;117:1016–1037. 1443971 10.7326/0003-4819-117-12-1016

[7] Rossouw JE, Anderson GL, Prentice RL, LaCroix AZ, Kooperberg C, Stefanick ML, et al Writing Group for the Women's Health Initiative Investigators. Risks and benefits of estrogen plus progestin in healthy postmenopausal women: principal results from the Women’s Health Initiative randomized controlled trial. JAMA 2002;288:321–333. 12117397 10.1001/jama.288.3.321

